# Arginine Is a Novel Drug Target for Arginine Decarboxylase in Human Colorectal Cancer Cells

**DOI:** 10.3390/ijms241813741

**Published:** 2023-09-06

**Authors:** Xinlei Wei, Ho-Yin Chow, Hiu-Chi Chong, Siu-Lun Leung, Mei-Ki Ho, Man-Yuen Lee, Yun-Chung Leung

**Affiliations:** 1 Department of Applied Biology and Chemical Technology, The Hong Kong Polytechnic University, Hung Hom, Kowloon, Hong Kong, China; 2 Lo Ka Chung Research Centre for Natural Anti-Cancer Drug Development, The Hong Kong Polytechnic University, Hung Hom, Kowloon, Hong Kong, China; 3 State Key Laboratory of Chemical Biology and Drug Discovery, The Hong Kong Polytechnic University, Hung Hom, Kowloon, Hong Kong, China

**Keywords:** arginine depletion, argininosuccinate synthetase, colorectal cancer, cell-cycle arrest, apoptosis, arginine decarboxylase

## Abstract

Colorectal cancer (CRC) has been proven to be highly reliant on arginine availability. Limiting arginine-rich foods or treating patients with arginine-depleting enzymes arginine deiminase (ADI) or arginase can suppress colon cancer. However, arginase and ADI are not the best drug candidates for CRC. Ornithine, the product of arginase, can enhance the supply of polyamine, which favors CRC cell growth, while citrulline, the product of ADI, faces the problem of arginine recycling due to the overexpression of argininosuccinate synthetase (ASS). Biosynthetic arginine decarboxylase (ADC), an enzyme that catalyzes the conversion of arginine to agmatine and carbon dioxide, may be a better choice as it combines both arginine depletion and suppression of intracellular polyamine synthesis via its product agmatine. ADC has anti-tumor potential yet has received much less attention than the other two arginine-depleting enzymes. In order to gain a better understanding of ADC, the preparation and the anti-cancer properties of this enzyme were explored in this study. When tested in vitro, ADC inhibited the proliferation of three colorectal cancer cell lines regardless of their ASS cellular expression. In contrast, ADC had a lesser cytotoxic effect on the human foreskin fibroblasts and rat primary hepatocytes. Further in vitro studies revealed that ADC induced S and G2/M phase cell-cycle arrest and apoptosis in HCT116 and LoVo cells. ADC-induced apoptosis in HCT116 cells followed the mitochondrial apoptotic pathway and was caspase-3-dependent. With all results obtained, we suggest that arginine is a potential target for treating colorectal cancer with ADC, and the anti-cancer properties of ADC should be more deeply investigated in the future.

## 1. Introduction

Arginine is a semi-essential amino acid that plays a pivotal role in many metabolic processes related to cell growth and proliferation, such as the biosynthesis of proteins and polyamines. It is more indispensable for cancer cells than for normal cells. Many tumor cells cannot produce arginine themselves via the urea cycle, making them vulnerable to arginine deprivation [1,2,3]. In an arginine-free medium (AFM), non-tumorous cells, including human fibroblasts, rat kidney epithelial cells, and mink lung epithelial cells, enter a quiescent state (G0 phase) that can last for several weeks. The cells can recover when the supplementation of arginine resumes; however, a wide variety of cancer cell lines, due to their defective cell-cycle checkpoints have a continuous cell division process which eventually leads to cancer-cell death [4]. These observations suggest that arginine auxotrophy may be the Achilles’ heel of tumor cells, and arginine-depleting enzymes are potential targeted therapeutic agents for cancer.

Enzymatic deprivation of arginine has become a promising anti-cancer strategy in recent years. Among all arginine-depleting enzymes, arginine deiminase (ADI) from mycoplasma is the best studied in terms of anti-cancer therapy. Pegylated ADI, namely ADI-PEG20, not only acts effectively against a variety of cancer cell lines deficient in argininosuccinate synthetase (ASS) [2,5,6,7], but also yields promising results in phase II/III studies in patients with hepatocellular carcinoma (HCC) [8,9] and melanoma [10,11]. Pegylated recombinant human arginase I (Peg-rhArg1) has also completed its phase I and II trials in patients with HCC, and has shown a good safety profile with significant efficacy [12,13]. Colorectal cancer has recently become the third most frequently diagnosed cancer in males and the second in females worldwide [14]; however, standard conventional chemotherapies for this type of cancer, such as FOLFOX (oxaliplatin plus 5-FU and leucovorin), FOLFIRI (irinotecan plus 5-FU and leucovorin), and XELOX (oxaliplatin plus capecitabine), frequently result in symptoms such as nausea, vomiting, diarrhea, neutropenia, febrile neutropenia, paresthesia, and dehydration [15]. Targeted therapy such as cetuximab, panitumumab, and bevacizumab have shown promise in treating colorectal cancer; however, they are expensive, and patients sometimes develop drug resistance [16]. Therefore, there is a real need to develop extra therapeutic agents for treating colorectal cancer.

Although researchers have proved that arginine depletion has an anti-cancer effect against CRC [17], using ADI and arginase in treating CRC may have some drawbacks. Arginine succinate synthetase (ASS) and argininosuccinate lyase (ASL), enzymes that catalyze arginine synthesis from citrulline, have been identified as upregulated targets in primary human colorectal tumors. As the metabolic product of ADI, citrulline can easily be recycled to arginine via the overexpressed ASS and ASL in the urea cycle, failing to generate a severe arginine-starving environment [18]. Arginase can induce strong arginine depletion; however, its product ornithine may enhance CRC progression. CRC is associated with elevated levels of ornithine decarboxylase (ODC) activity [19] which catalyzes the conversion of ornithine to polyamines. Polyamines are essential modulators of cellular proliferation, DNA synthesis, and differentiation due to their ability to regulate transcription, translation, DNA methylation, and signal transduction pathways [20,21]. The formation of ornithine by arginase and the elevated ODC level may cause a boost in polyamine levels in tumor cells [22].

The discovery of ADC dates back to the 1960s [23]. Historically, arginine decarboxylase (ADC) was recognized as a product of plants, bacteria, and invertebrates. However, subsequent discoveries revealed the presence of measurable levels of agmatine and ADC activity in the brains of humans, rats, and bovines. Besides, ADC is also expressed in several other organs, albeit the extent of its expression is considerably limited [24]. The anti-cancer properties of this enzyme have long been overlooked despite a research team mentioning the inhibitory effect of ADC on HeLa cells in vitro [25]. ADC is an arginine-depleting enzyme that converts L-arginine to agmatine and carbon dioxide. It has a dual effect against CRC. Besides inducing arginine deprivation, its metabolic product agmatine is a potent ODC inhibitor that can reduce the intracellular polyamine level. Blocking polyamine synthesis using ODC inhibitor difluoromethylornithine (DFMO) in CRC cell lines has been shown to inhibit cell proliferation and increase apoptosis, proving the importance of suppressing ODC in CRC treatment [26]. Moreover, agmatine can escape the arginine recycling process via ASS, which is better than the citrulline generated by ADI. In the present study, we tested the effect of arginine deprivation on colorectal cancer cells with recombinant biosynthetic ADC from *E. coli*. We demonstrated that ADC is much more cytotoxic against colorectal cancer cells than non-tumorous cells and is, surprisingly, a potent apoptosis inducer for colorectal cancer cells. Our results revealed the potential weakness of colorectal cancer cells and the possibility of ADC being developed as a targeted therapeutic agent against this type of cancer.

## 2. Results

### 2.1. A Single-Step Purification of Recombinant ADC Resulted in High Protein Yield

**Figure 1:**
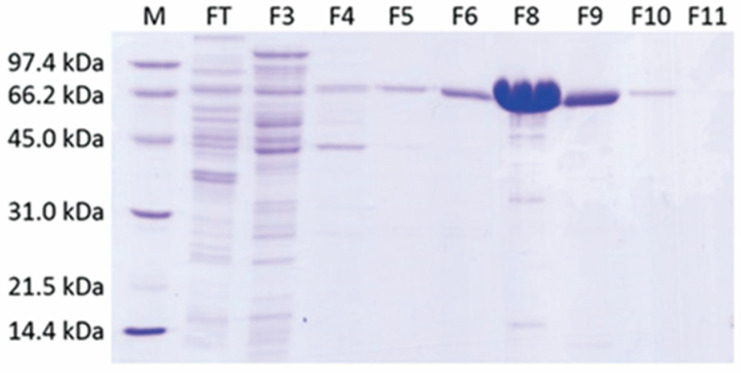
SDS-PAGE analysis of the column fractions from nickel affinity chromatography for the 6xHis-tagged ADC. M, SDS-PAGE molecular weight standards, low range (Bio-Rad); FT, flow through; F3-F11, eluted fractions from the column.

His-tagged ADC was produced using the *E. coli* expression system. The cell lysate of *E. coli* was then centrifuged and soluble protein was subjected to a single-step purification with affinity chromatography, and active fractions were eluted at 0.3 M imidazole. These fractions were pooled and exchanged buffer for formulation. The powerful T7 promoter resulted in a high level of ADC expression, and more than 100 mg of ADC was purified from 1 L bacterial culture grown in a shake flask (Table 1). According to SDS-PAGE analysis, molecular size of ADC was around 66 kDa, and nickel affinity chromatography provided a simple yet effective separation of ADC from impurities (Figure 1). Specific activity of purified ADC was 27 units/mg and was stable for at least six months of storage.

### 2.2. ADC Was Better Than ADI in Treating ASS-Positive Colorectal Cancer Cells

**Figure 2:**
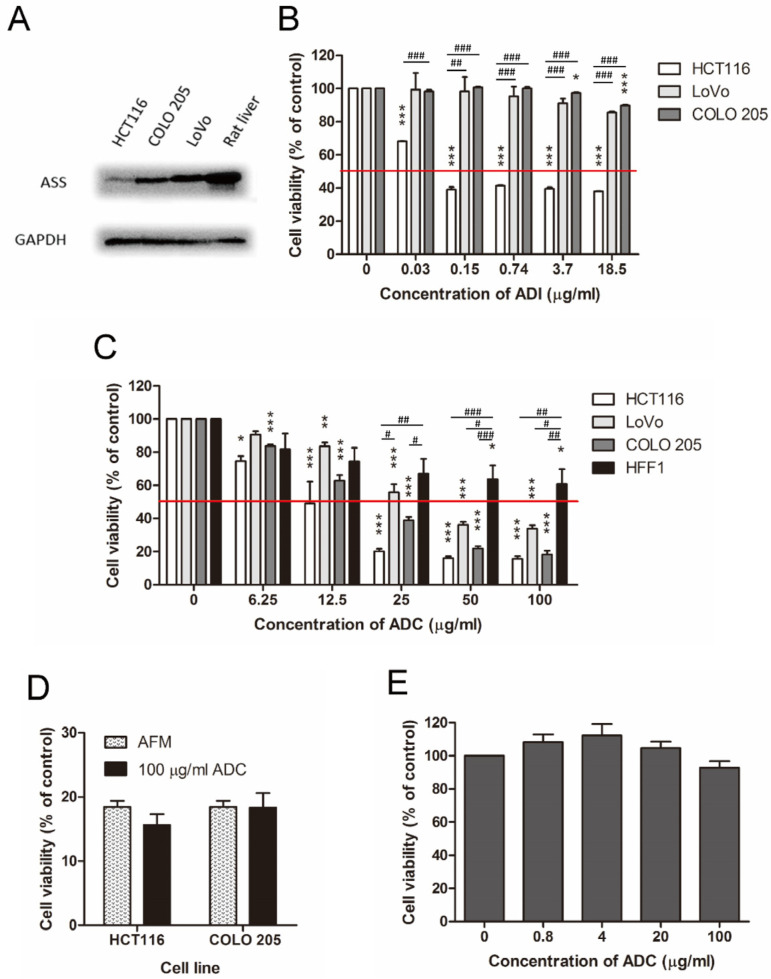
ADC performed better than ADI in ASS-positive human colorectal cancer cell lines and was less toxic to normal cells. (**A**) Immunoblot analysis showing the expression of ASS in colorectal cancer cells. (**B**) Cell viability towards ADI of different cancer cell lines. (**C**) Cell viability towards ADC of different cancer cell lines and normal human fibroblast HFF1. (**D**) Bar chart comparing the effects of AFM and 100 μg/mL ADC on the viability of HCT116 and COLO 205 cells. Cells were treated for 72 h before MTT analysis. (**E**) Cell viability towards ADC in rat primary hepatocytes. Data are expressed as the percentage of viable cells compared to control (complete medium) in the form of mean ± SEM of three individual experiments. * *p* < 0.05, ** *p* < 0.01, *** *p* < 0.001 using Mann–Whitney U test (versus control). ^#^ *p* < 0.05, ^##^ *p* < 0.01, ^###^ *p* < 0.001 using one-way ANOVA followed by Bonferroni test (between groups).

Three ASS-positive human colorectal cancer cell lines including HCT116, LoVo, and COLO 205 (Figure 2A), were tested for their sensitivity to arginine depletion induced by ADI and ADC. After 72 h of drug treatment, cell viability was determined by MTT assay. Consistent with the widely-accepted conclusion, cells sensitivity to ADI was correlated with the lack of intracellular ASS expression [1,2,3,5,7]. We observed that HCT116 cells expressed a lower level of ASS and was more ADI-sensitive, while LoVo and COLO 205 cells both had stronger ASS expression and were more ADI-resistant (Figure 2A,B). ADC treatment resulted in notable dose-dependent inhibition in all three colorectal cancer cell lines (Figure 2C), with IC50 values ranging from 12.2 to 38.1 μg/mL (Table 2).

When used at a high concentration (100 μg/mL), the growth-inhibitory effect of ADC was similar to that of AFM (Figure 2D), suggesting that arginine depletion may be the main anti-proliferative mechanism of ADC on these cell lines. Yet, we also observed that agmatine, the catalytic product of ADC, showed slight anti-proliferative effect on colorectal cancer cell lines. According to our measurement, IC50 of agmatine was 1.8 mM in HCT116 cells and 1.9 mM in COLO205 cells.

### 2.3. ADC Was Relatively Less Toxic to Non-Tumorous Cells

In contrast to tumor cells, the ASS-positive human foreskin fibroblast cell line HFF1 [27] was more resistant to ADC treatment. Incubation of HFF1 with 100 μg/mL of ADC resulted in more than 50% viable cells after 72 h incubation (Figure 2C). When tested in rat primary hepatocytes, ADC was found to have negligible inhibitory effect on cell viability (Figure 2E).

### 2.4. ADC Induced S and G2/M Arrest in Colorectal Cancer Cells

**Figure 3:**
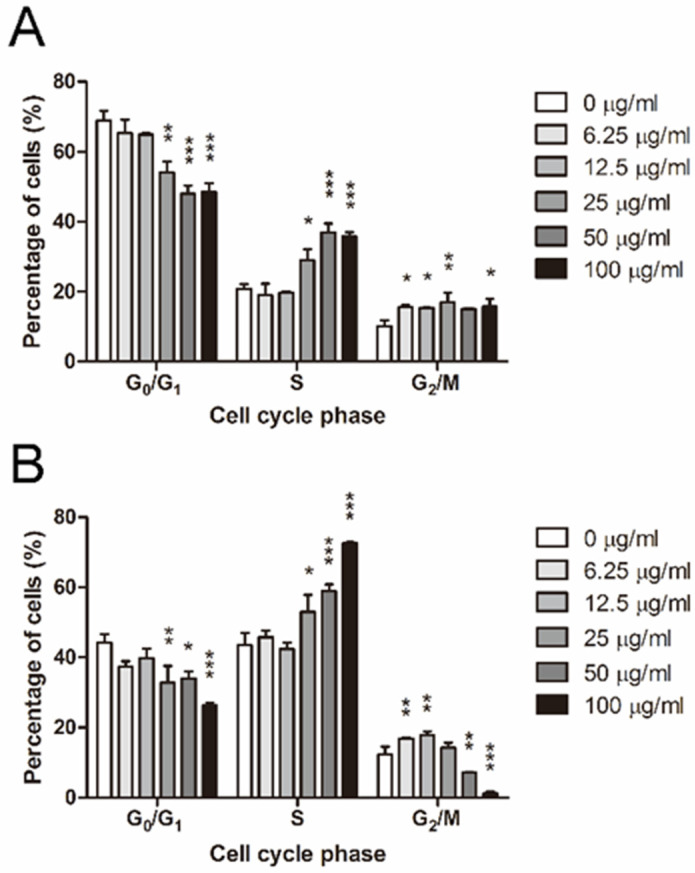
HCT116 and LoVo cells were exposed to different concentrations of ADC for 72 h and analyzed by flow cytometry for their cell-cycle profiles. (**A**) Cell-cycle profile in HCT116 cells. (**B**) Cell-cycle profile in LoVo cells. Data are expressed as mean ± SEM of three individual experiments. * *p* < 0.05, ** *p* < 0.01, *** *p* < 0.001 using Mann–Whitney U test (versus control).

To investigate the effect of ADC on cell-cycle progression, HCT116 and LoVo cells were exposed to different concentrations of ADC for 72 h and analyzed by flow cytometry for their cell-cycle profiles. In HCT116 cells, the S phase subpopulation remained steady at low doses of ADC, but rose at 25, 50, and 100 μg/mL ADC in a dose-dependent manner (Figure 3A). The proportion of G2/M phase slightly increased in response to ADC treatment and peaked at 25 μg/mL ADC (Figure 3A). LoVo cells responded to ADC in a slightly different manner from HCT116 cells by arresting at G2/M phase under the exposure to low doses of ADC but switching to S phase arrest when the dose of ADC was further increased (Figure 3B).

### 2.5. ADC Induced Apoptosis in Colorectal Cancer Cells in a Time- and Dose-Dependent Manner

**Figure 4:**
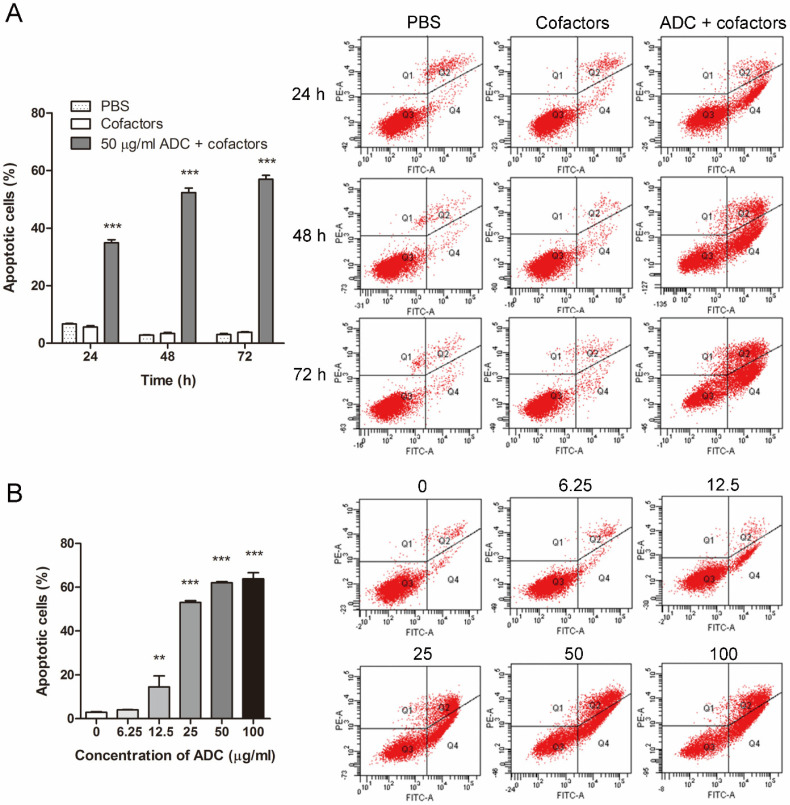
ADC induced apoptosis in colorectal cancer cells in a time- and dose-dependent manner. (**A**) Bar chart showing the apoptosis percentage in HCT116 cells after 24, 48, and 72 h of treatment with ADC. (**B**) Bar chart showing the apoptosis percentage in HCT116 cells after 72 h of treatment with different concentrations of ADC. Data are expressed as mean ± SEM of three individual experiments. ** *p* < 0.01, *** *p* < 0.001 using Mann–Whitney U test (versus control).

Previous research has indicated that arginine starvation can induce apoptosis in cells, triggering stress responses due to the absence of arginine. This not only leads to cell cycle arrest, but also activates pathways leading to apoptosis [28,29,30]. As such, we have further investigated the specific apoptosis pathway induced by ADC. Flow cytometry results suggested that apoptosis was induced in HCT116 cells in a time-dependent manner upon ADC treatment. At 24, 48, and 72 h post-ADC-treatment, ~35%, 52%, and 57% of cells underwent apoptosis, respectively (Figure 4A). The presence of cofactors alone (5 mM MgCl_2_ and 0.1 mM PLP) had no effect on either the necrosis or apoptosis status of cells. In contrast, active ADC in the presence of cofactors induced apoptosis in HCT116 cells in a dose-dependent manner. After 72 h of treatment, compared to the control group (0 μg/mL ADC, 2.93% cells underwent apoptosis), the apoptotic cell population remained relatively unchanged at 6.25 μg/mL ADC (4% cells underwent apoptosis), but increased dramatically 4.9-, 18.1-, 21.2-, and 21.8-fold at 12.5, 25, 50, and 100 μg/mL ADC, respectively (Figure 4B).

### 2.6. ADC-Induced Apoptosis in HCT116 Cells Was Caspase-3-Dependent

**Figure 5:**
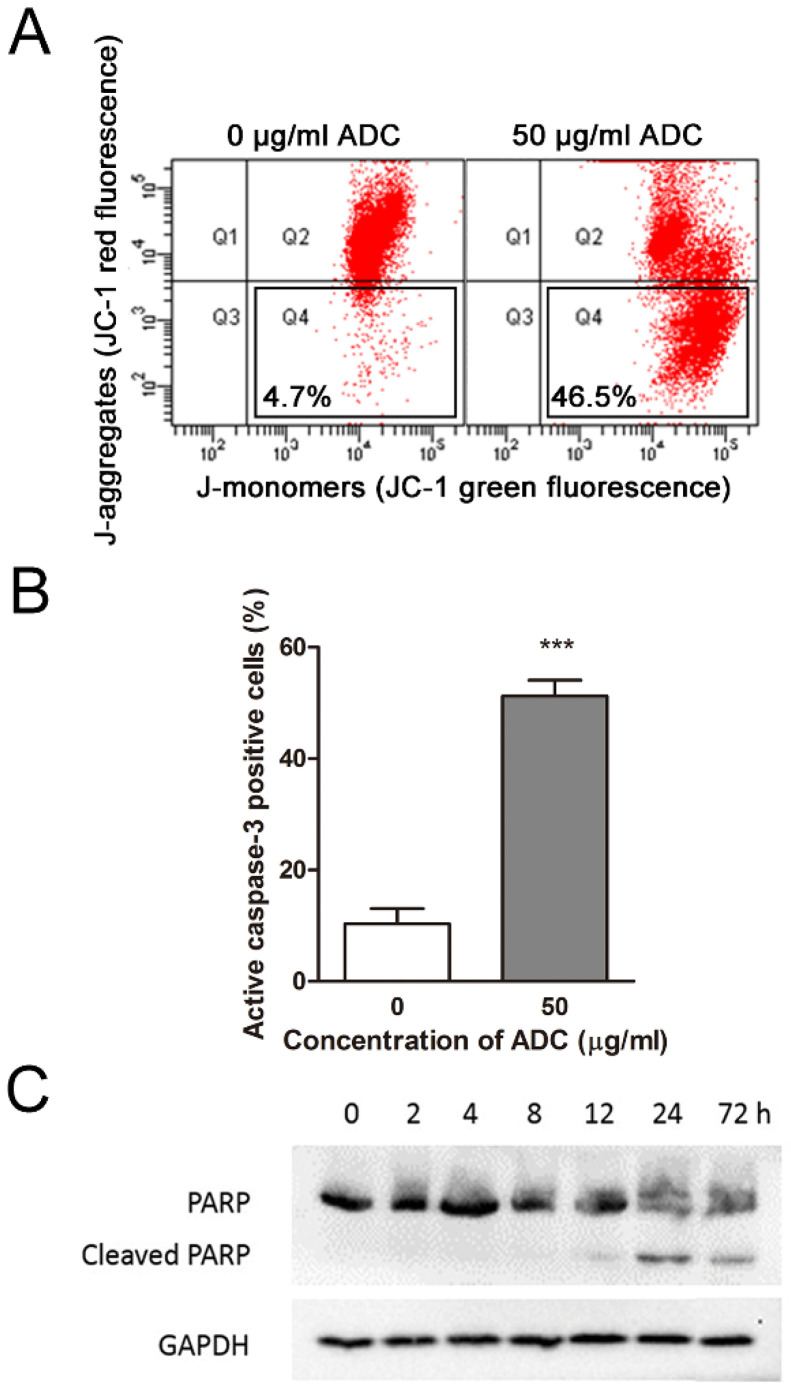
ADC-induced apoptosis in HCT116 cells was caspase-3-dependent. (**A**) Representative flow cytometry data from triplicated experiments showing changes in MOMP in HCT116 cells using JC-1 dye. Cells were grown for 72 h prior to analysis. (a’) control; (b’) 50 μg/mL ADC. (**B**) FITC-DEVD-FMK staining and flow cytometry results showing the percentage of HCT116 cell population with active caspase-3 upon ADC treatment. Data are expressed as mean ± SEM of three individual experiments. *** *p* < 0.001 using Mann–Whitney U test (versus control, 0 μg/mL ADC). (**C**) Immunoblot analysis showing the time-dependent effect of 50 μg/mL ADC on the cleavage of PARP in HCT116 cells.

The mechanism of ADC-induced apoptosis in HCT116 cells was then exploited. HCT116 cells were treated with 50 μg/mL ADC for 72 h, stained with JC-1, and analyzed by flow cytometry. As a cationic dye, JC-1 accumulates potential-dependently in the form of red fluorescent aggregates in mitochondria, and dissociates into green fluorescent monomers when diffused into cytosol due to change in electrochemical gradient of mitochondrial outer membrane (MOM). The decrease in red/green fluorescence intensity ratio in ADC-treated HCT116 cells indicated the depolarization of mitochondria and the increase in mitochondrial outer membrane permeabilization (MOMP). The percentage of ADC-treated HCT116 cells with increased MOMP was ~47% (Figure 5A) which was quite comparable to the apoptotic population, suggesting that a major portion of the HCT116 cell population died due to the mitochondrial apoptotic pathway upon ADC treatment.

As the release of mitochondrial contents can activate caspases to execute apoptosis, we then determined whether caspases had been activated by focusing on caspase-3, a typical caspase in apoptotic study. Consistent with the cell population undergoing apoptosis as well as the population with increased MOMP, ~47% of HCT116 cells became caspase-3-activated when treated with 50 μg/mL of ADC for 72 h (Figure 5B). Further examination of the cleavage of poly (ADP-ribose) polymerase (PARP), a known substrate of caspase-3, showed that cleavage of PARP was invisible until 12 h after ADC treatment (Figure 5C), suggesting ADC-induced apoptosis in HCT116 cells is caspase-3-dependent.

## 3. Discussion

The utilization of enzymes as anti-cancer drugs is a relatively novel approach compared with the application of other targeted therapeutic agents such as small-molecule inhibitors and monoclonal antibodies. The most successful example of this approach is asparaginase, an enzyme that depletes asparagine, which is used against leukemia [31,32]. Native asparaginase from *E. coli* and Erwinia chrysanthemi was approved by the US Food and Drug Administration (FDA) in 1994 and 2011, respectively, for use in patients with acute lymphoblastic leukemia (ALL). The success of asparaginase in the treatment of leukemia has demonstrated the effectiveness of amino-acid deprivation against certain types of cancer, and revealed the hopeful prospect of native bacterial enzymes being developed into therapeutic agents for humans.

In this study, we prepared recombinant *E. coli* biosynthetic ADC from a powerful bacterial expression system (Figure 1). Compared to ADI, a well-studied arginine-depleting enzyme that requires multiple steps for purification with a protein yield of 1 mg per 1 L *E. coli* culture [33] or 3.6 mg per 250 mL *E. coli* culture (in this study), recombinant ADC is expressed at a much higher level in *E. coli* (100 mg per L cell culture, Table 1) in the form of soluble protein and is readily purified with a nickel affinity column. The time- and cost-effective preparation process of ADC makes it a promising drug material.

Our study showed that ADC inhibited the cell proliferation of several human colorectal cancer cell lines (Figure 2B and Supplementary Table S1) via cell-cycle arrest (Figure 3) but exerted less inhibitory effects on human foreskin fibroblasts (Figure 2C) and rat primary hepatocytes (Figure 2E). Our results suggest that colorectal cancer cells may have a higher demand for exogenous arginine than normal cells and hence are relatively vulnerable to arginine deprivation.

Agmatine, the catalytic product of ADC, is an anti-proliferative agent [34,35,36,37]. A study has shown that agmatine accumulates in colorectal cancer cells without being metabolized, and inhibits cell proliferation by reducing cellular polyamine levels [37]. Consistent with these findings, we observed that agmatine exerted an inhibitory effect on colorectal cancer cells with IC50 values of around 2 mM. Nevertheless, the cell-killing effect of a high dose of ADC in colorectal cancer cells may be mainly due to the deprivation of arginine, as similar inhibitory effects were achieved by both 100 μg/mL ADC and AFM (Figure 2D).

**Figure 6:**
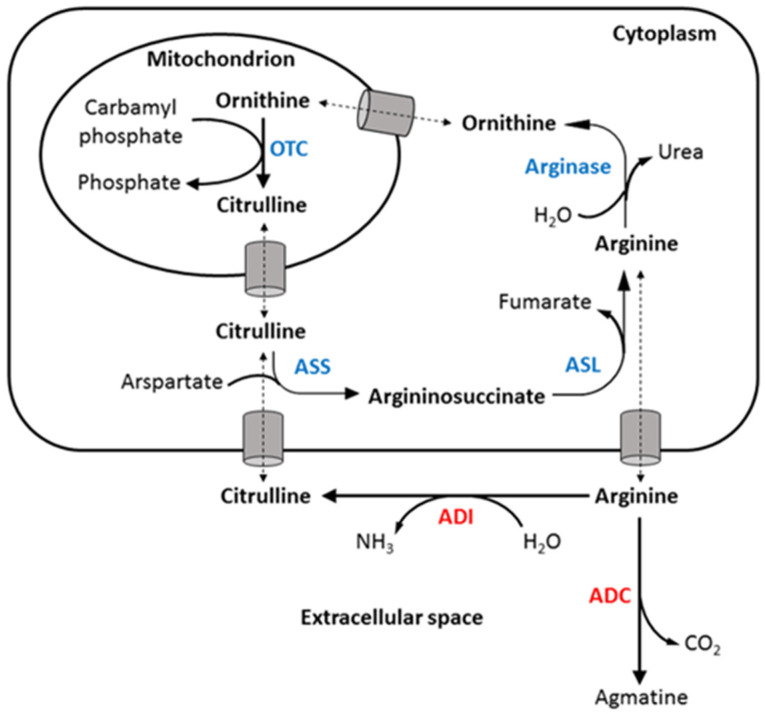
The difference in the performance of ADI and ADC is due to their distinct catalytic products. Unlike ADI which produces a urea-cycle intermediate (citrulline), the catalytic product of ADC (agmatine) is not involved in the urea cycle and thus, would not contribute to the de novo synthesis of arginine regardless of the level of ASS in cancer cells.

Cellular ASS, either constitutive or inducible, helps to convert citrulline to arginine and hence results in the resistance of tumor cells to ADI treatment [38,39,40]. As a result, melanoma and HCC, which are usually ASS-negative, are the main focus of arginine deprivation by ADI. On the contrary, colorectal cancers are generally of high ASS levels [18] and have not been explored much by studies of ADI so far. It should be noted that ASS is the rate-limiting enzyme in the urea cycle [40]. A low cellular level of ASS may not be sufficient to maintain cell growth through the de novo synthesis of arginine from citrulline [41]. Consistent with these findings, we observed that HCT116 cells express a low level of ASS (Figure 2A) and is an ADI-sensitive cell line, while LoVo and COLO 205 cells have higher levels of ASS and are ADI-resistant (Figure 2B). On the other hand, all these three cell lines are sensitive to ADC (Figure 2C). The difference in the performance of ADI and ADC is due to their distinct catalytic products. Unlike ADI, which produces a urea-cycle intermediate, citrulline, the catalytic product of ADC (agmatine), is not involved in the urea cycle and thus would not contribute to the de novo synthesis of arginine, regardless of the level of ASS in cancer cells (Figure 6). We tested the addition of 1.15 mM citrulline together with ADC to cell lines with varying levels of ASS expression, specifically ASS-low HCT116 and ASS-high COLO205 cells. In a standard culture medium devoid of citrulline, both cell lines exhibited significant cytotoxicity when treated with ADC alone (Supplementary Figure S1A,B). However, the introduction of extra citrulline led to an improvement in cell viability solely in the ASS-high COLO205 cells (Supplementary Figure S1B). Therefore, ADC seemed to have a broader anticancer spectrum than ADI based on their difference in catalytic product.

Ornithine transcarbamylase (OTC) is another crucial enzyme in the urea cycle, which, together with ASS, is responsible for the resistance of tumors to arginase [42]. Theoretically, regarding arginine recycling, ADC is advantageous over arginase in treating cancers as it should be independent of the tumorous level of urea cycle enzymes. However, this hypothesis needs to be tested, since all three colorectal cancer cell lines in this study showed undetectable levels of OTC protein. As the cellular expression of OTC seems less commonly observed in tumors than the expression of ASS [42,43], both ADC and arginase are likely to be of considerable value in future cancer treatment. Furthermore, ADC may be a potential alternate anti-cancer agent to arginase if any resistance to the latter is developed during treatment.

The observation that arginine deprivation induces apoptotic cell death in cancer cells has been reported by multiple groups of researchers studying ADI and arginase [6,43,44]. Based on these findings, we evaluated the pro-apoptotic effect of ADC in colorectal cancer cells (Figure 4). We found that ADC was a robust apoptotic inducer in the two cell lines tested, as a 3-day treatment of 50 μg/mL ADC resulted in ~60% of cells undergoing apoptotic cell death.

The mechanism of ADC-induced apoptosis in HCT116 cells was then studied. Based on the reported findings that leucine deprivation causes cell death through the mitochondrial apoptotic pathway [45], we hypothesized that the lack of arginine may also act as a stimulus of apoptosis by increasing the permeability of mitochondria and may hence cause the release of pro-apoptotic proteins into the cytosol. A study has reported that ADI can reduce the MOMP in the ASS-deficient breast cancer cell line MDA-MB-231 [7]. A similar finding was derived in our study with ADC in HCT116 cells (Figure 5). While we observed cell-cycle arrest (Figure 3B) and apoptosis (Supplementary Figure S2A) in LoVo cells, unlike HCT116 cells, the induced apoptosis in LoVo cells was not dependent on caspase-3 (Supplementary Figure S2B). This variation in apoptosis induction may originate from the inherent heterogeneity of cancer cells. To unravel the specific cell-death pathways across all types of CRC, further experiments are necessary.

The effect of ADC on cell-cycle progression was also examined, as cell-cycle disruption is another possible reason for the observed attenuation of cell growth. The S and/or G2/M phase arrest was not unique to ADC-treated colorectal tumor cells (Figure 3). Concomitantly, there are reports that ADI induces G0/G1 cell-cycle arrest in cell lines such as stomach adenocarcinoma (SNU-1), neuroblastoma (SH-EP), and leukemia (DU145) [46,47] S phase arrest is found in some other cell lines, including those of neuroblastoma (WAC2), osteosarcoma (SaOS), and leukemia (Jurkat) [48,49]. S and G2/M arrest was also detected in HCC cell lines treated with pegylated recombinant human arginase I (rhArg-PEG) [50]. Therefore, ADC may share similar anti-cancer mechanisms with ADI and arginase, as all three enzymes deprive arginine.

We employed TCGA (The Cancer Genome Atlas) database, to analyze the expression levels of ASS in colon adenocarcinoma (COAD) and other cancer types (Supplementary Figure S3). The results indicated that cancers sensitive to ADI, such as cholangiocarcinoma (CHOL) (Supplementary Figure S3A) [51] and liver hepatocellular carcinoma (LIHC) (Supplementary Figure S3B) [52], exhibit significant downregulation of ASS levels. However, in contrast to these cancers, COAD did not show this downregulation of ASS (Supplementary Figure S3C). This observation further supports the notion that the current arginine-depleting drug, ADI, may not be effective against colorectal cancer due to the potential recycling of arginine from its product, citrulline, via ASS. In contrast, ADC, proposed in our manuscript, could be a more suitable candidate, as its product, agmatine, cannot be converted back to arginine, thus creating an arginine auxotrophic environment within cancer cells. 

The application of ADC in vivo presents challenges. Our preliminary data shows that an intravenous injection of 2.5 mg ADC per mice can successfully deplete circulating arginine levels within 2 h. However, the half-life of ADC in its native form is not sufficiently long to consistently maintain an arginine-deprived environment in vivo. Future efforts need to focus on modifying the protein and adjusting the formulation to extend the drug’s half-life, thereby enhancing its potential therapeutic effectiveness. In summary, our results have demonstrated the importance of arginine for the growth and proliferation of colorectal cancer cells and suggest that ADC, an arginine-depleting enzyme, is a potential targeted therapeutic agent against this type of cancer.

## 4. Materials and Methods

### 4.1. Construction of Expression Vector for ADC

The expression vector for ADC was constructed as follows: the DNA sequences encoding ADC were amplified from *E. coli* DH5α by the polymerase chain reaction with forward and reverse primers containing recognition sites for restriction enzymes NdeI and BamHI, respectively (underlined):

(a) 5′-GCATATGAGCAAGATGCTGCGTACTTACAATATTGCCTGGTGGGGC-3′
(b) 5′-GCAGCCGGATCCTTAGTGATGGTGGTGGTGGTGCTCATCTTCA-3′

Site-directed mutagenesis of ADC was performed by PCR to change the 1299th base pair of T into C, thereby silencing the internal BamHI site in ADC. The forward and reverse primers used were as follows:

(c) 5′-GAAGTGCAAAAGCAGCTGGACCCGCAAAACCG-3′
(d) 5′-CGGTTTTGCGGGTCCAGCTGCTTTTGCACTTC-3′

The PCR product was digested with NdeI and BamHI, ligated into a pET-3a vector, and transformed into *E. coli* BL21(DE3) cells. This construction resulted in a sequence of a matured form of ADC (with its first 32 amino acid residues representing the signaling peptide sequence removed) with a methionine residue at the N-terminus and a 6xHis-tag at the C-terminus.

### 4.2. Preparation of ADC

The bacterial stock that expressed ADC was inoculated into 10 mL of LB medium containing 100 μg/mL ampicillin, and grown overnight at 37 °C with shaking. The cell culture was then added to 250 mL of fresh LB medium at a volume ratio of 1:100, and grown at 37 °C with shaking. When the absorbance of the culture at 600 nm reached 0.6–0.8, the expression of ADC was induced by adding 0.2 mM isopropyl β-D-1-thiogalactopyranoside (IPTG) to the culture. After an additional 4 h of incubation, a cell pellet was collected by centrifugation and was resuspended in buffer containing 50 mM Tris-HCl, 100 mM NaCl, and 5 μM MgCl_2_, at pH 7.4. The suspended cells were sonicated on ice. The lysate obtained was centrifuged at 10,000 rpm for 30 min to remove the insoluble contents. ADC was purified by a HiTrapTM chelating column (GE Healthcare) charged with Ni2+ and eluted with a gradient of 0–0.5 M imidazole using an ÄKTA purifier (GE Healthcare). Purified ADC was formulated at 4–5 mg/mL in 20 mM sodium phosphate buffer (pH 7.4) supplemented with 5 mM MgCl_2_, 0.1 mM PLP, and stored at 4 °C in darkness.

### 4.3. Specific Activity of ADC

To determine the enzyme activity, ADC was incubated at 37 °C, pH 8.0 with excessive L-arginine as substrate. A subsequent reaction with *E. coli* agmatinase at 45 °C, pH 9.0 was carried out to convert agmatine produced by the ADC-catalyzed reaction to putrescine and urea. The amount of urea was determined through the diacetyl monoxime (DAMO) method which spectrophotometrically quantifies urea in samples. One unit of ADC is defined as the amount of enzyme that eventually produces 1 μmol urea per min at 37 °C, pH 8.0. Protein concentration was estimated by Bradford assay using protein assay dye reagent concentrate (Bio-Rad), with bovine serum albumin (Amresco) as the standard.

### 4.4. Preparation of ADI

Recombinant mycoplasma ADI expressed in *E. coli* was prepared as described previously [53]. The enzyme activity of ADI was assayed by directed colormetric method [54]. From 250 mL shake flask culture, around 3.6 mg of ADI was purified and the specific activity was 42.6 units/mg. One unit of ADI was defined as the amount of enzyme that produces 1 μmol citrulline per min at 37 °C, pH 7.5.

### 4.5. Culture of Cell Lines

Human colorectal carcinoma cell lines HCT116 and LoVo cells, and human colorectal adenocarcinoma cell line COLO205 were purchased from the National Cancer Institute (NCI, Rockville, MD, USA). Human foreskin fibroblast cell line HFF1 was purchased from the American Type Culture Collection (Manassas, VA, U.S.). All tumor cells were maintained in RPMI 1640 medium (Invitrogen, Waltham, MA, U.S.) supplemented with 10% (*v*/*v*) fetal bovine serum (FBS; Hyclone) and 100 units/mL penicillin/streptomycin (P/S; Invitrogen). HFF1 cells were grown in DMEM medium (Invitrogen) supplemented with 15% (*v*/*v*) FBS and 100 units/mL P/S. All cells were cultured at 37 °C with 5% CO_2_, 90% humidity.

### 4.6. Preparation and Culture of Rat Primary Hepatocytes

Rat primary hepatocytes were isolated by a two-step collagenase perfusion technique from male Sprague Dawley rats. Rats were obtained from the Laboratory Animal Service Centre, The Chinese University of Hong Kong. Rats were housed in a 12 h light-dark cycle holding room with free access to rat chow (Glen Forrest Stockfeeders, Glen Forrest, Australia) and tap water. All the experimental procedures were approved by the Animal Subject Ethics Committee (POLYU) in strict accordance with the animal license approved by the Department of Health (HKSAR, Ref No.: (22-396) in DH/HT&A/8/2/4 Pt.12).

### 4.7. Cell Viability Assay

Cell viability was evaluated by the MTT assay. For all cell lines, 5 × 10^3^ cells in 100 μL of culture medium were seeded to each well of a 96-well plate and incubated for 24 h. For rat primary hepatocytes, 5 × 10^3^ freshly isolated cells were seeded for 3 h in DMEM supplemented with 10% (*v*/*v*) FBS, 1% (*v*/*v*) P/S and antimycotics, and 100 nM dexamethasone. The medium was then replaced by DMEM supplemented with 0.1% (*v*/*v*) BSA, 1% (*v*/*v*) P/S and antimycotics, and 100 nM dexamethasone and incubated for another 21 h. The culture medium was then replaced with fresh culture medium with varying concentrations of ADC, ADI, or agmatine sulfate (Sigma, Marlborough, MA, USA), or by arginine-free medium (AFM; United States Biological, Salem, MA, USA). For the treatment of ADC, culture medium was also supplemented with 1 mM MgCl_2_ and 0.1 mM PLP. Cells were then incubated for another 3 days. At the end of the treatment period, 10 μL of 5 mg/mL 3-(4,5-dimethylthiazol-2-yl)-2,5-diphenyltetrazolium bromide (MTT; Invitrogen) reagent was added to each well and the plate was incubated for another 4 h. MTT is reduced to formazan by viable cells. To dissolve the produced formazan, 100 μL of 10% SDS, and 0.01 N HCl was added to each well, and the plate was incubated overnight before the absorbance at 570 nm was measured. Non-linear regression with Prism 8.0.2 (Graphpad Software, Boston, MA, USA) was used to fit a sigmoidal dose-response curve. The amount of ADC required to achieve 50% inhibition of cell viability was defined as IC50. To examine the effect of ASS on cell viability, 1.15 mM citrulline was added together with various concentrations of ADC.

### 4.8. Cell-Cycle Analysis

Cell-cycle distribution was assessed by flow cytometry with PI staining. For this, 105 cells per well were seeded in a 6-well plate, grown overnight, and treated with various doses of ADC in medium containing 1 mM MgCl_2_ and 0.1 mM PLP for 72 h. Cells were then collected by trypsinization, fixed in 75% ice-cold ethanol, washed in PBS, stained in PI/RNase Staining Buffer (BD Biosciences, San Jose, CA, USA) at room temperature for 15 min in the dark, and analyzed by flow cytometry (FACSAria, BD Biosciences). For each sample, data from 104 cells were collected and analyzed with ModFit LT 3.0 (Verity Software House).

### 4.9. Apoptosis Analysis by Flow Cytometry

In this step, 105 cells per well were seeded in a 6-well plate, grown overnight, and treated with various doses of ADC in medium containing 1 mM MgCl_2_ and 0.1 mM PLP for the desired amount of time. Cells were then collected by trypsinization, washed in PBS, and subjected to different staining with distinct purposes, as described by the manufacturers. Evaluation of apoptotic cell population was achieved with the Annexin V-FITC Apoptosis Detection Kit (BD Biosciences). Briefly, cells were stained in binding buffer (10 mM HEPES/NaOH, 140 mM NaCl, and 2.5 mM CaCl_2_, pH 7.4) containing Annexin V-FITC and PI at room temperature for 15 min in the dark before analysis. The detection of changes in mitochondrial outer membrane permeabilization (MOMP) was achieved by staining the cells with 1 μL JC-1 (Invitrogen) dissolved in 500 μL medium at 37 °C, 5% CO_2_ for 10 min, and washing twice in PBS before analysis. The cell population with active caspase-3 was determined using the CaspGLOW Fluorescein Active Caspase-3 Staining Kit (BioVision). Basically, cells were resuspended in 300 μL pre-warmed medium containing 1 μL FITC-DEVD-FMK, incubated at growth condition for 30 min, and washed twice with wash buffer before analysis. All analyses were performed in a FACSAria flow cytometer.

### 4.10. Immunoblot Assay

For the detection of ASS, 3 × 10^6^ cells were collected by trypsinization, washed in PBS, and lysed in RIPA buffer (Millipore). For the detection of PARP cleavage, 106 cells per well were seeded in a 6-well plate, grown overnight, treated with 50 μg/mL of ADC in medium with 1 mM MgCl_2_ and 0.1 mM PLP for 0, 2, 4, 8, 12, 24, and 72 h prior to harvest. Proteins in cell lysates were separated by SDS-PAGE, and specific bands were visualized by Western blot using ASS, PARP, and GAPDH antibodies (Cell Signaling Technology, Danvers, MA, U.S.). Horseradish-peroxidase-conjugated goat anti-rabbit IgG (Cell Signaling Technology) was used as a secondary antibody for enhanced chemiluminescence.

### 4.11. Statistical Analysis

For all statistical analyses, data were analyzed by Prism 5.0 using either two tailed t-test or one-way analysis of variance (ANOVA) with post hoc Dunnett’s multiple comparison test. *p* < 0.05 was considered significantly different.

## Figures and Tables

**Table 1:** Purification of recombinant *E. coli* biosynthetic ADC from 1 L shake flask culture.

Stage	Total Enzyme Activity (U)	Total Protein (mg)	Specific Enzyme Activity (U/mg)	Fold Purification	Recovery (%)
Whole cell lysate	4113.5	304.7	13.5	1.00	100.00%
Soluble proteins	3757.0	221.0	17.0	1.26	91.33%
Affinity column pooled fractions	3144.1	111.1	28.3	2.10	76.43%
Final Product	3005.7	110.1	27.3	2.02	73.07%

**Table 2:** IC50 of ADC in cell lines. IC50 value is defined as the amount of ADC needed to achieve 50% inhibition of cell growth.

Cell Types	Cell Lines	IC50 of ADC (μg/mL)
	HCT116	12.23
Human colorectal cancer	COLO205	19.40
	LoVo	38.09
Human foreskin fibroblast	HFF	>100
Rat primary hepatocyte	-	>100

## Data Availability

Not applicable.

## Supplementary Materials

The supporting information can be downloaded at: https://www.mdpi.com/article/10.3390/ijms241813741/s1.
