# Supplementary material for: Arginine Is a Novel Drug Target for Arginine Decarboxylase in Human Colorectal Cancer Cells
Source: Int J Mol Sci. 2023 Sep 6;24(18):13741. doi: 10.3390/ijms241813741 (PMC10531272; doi:10.3390/ijms241813741)
Supplement: Supplementary file 1 [file ijms-24-13741-s001.zip › ijms-2557001-supplementary.pdf]

Arginine Is a Novel Drug Target for Arginine Decarboxylase in Human Colorectal Cancer Cells

Xinlei Wei, Ho-Yin Chow, Hiu-Chi Chong, Siu-Lun Leung, Mei-Ki Ho, Man-Yuen Lee and Yun-Chung Leung

Supplementary Figure S1 Addition of citrulline can reverse the cytotoxic effect of ADC in ASS-high COLO205 cells but not ASS-low HCT116 cells. Cell viability towards ADC or ADC+citrulline on (A) COLO205 cells and (B) HCT116 cells after 72 h treatment. Data are expressed as mean ± SEM of three individual experiments. *p < 0.05, **p < 0.01 using Mann Whitney U test (versus control).

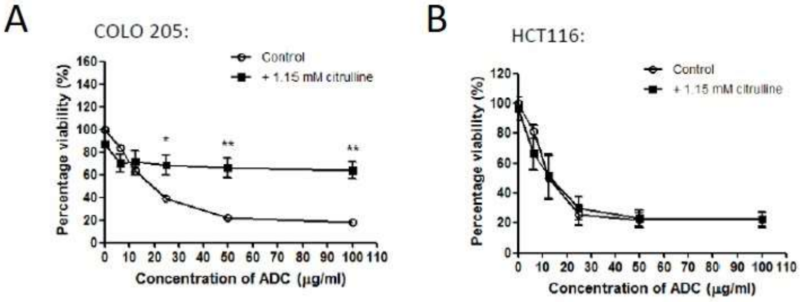

Supplementary Figure S**2** LoVo cells demonstrates a caspase 3-independent apoptosis upon ADC treatment. (A) The apoptosis percentage in LoVo cells after 72 h of treatment with different concentration of ADC. (B**)** FITC-DEVD-FMK staining and flow cytometry results showing the percentage of LoVo cell population with active caspase-3 upon ADC treatment. Data are expressed as mean ± SEM of three individual experiments. **p < 0.01, ***p < 0.001 using Mann Whitney U test (versus control).

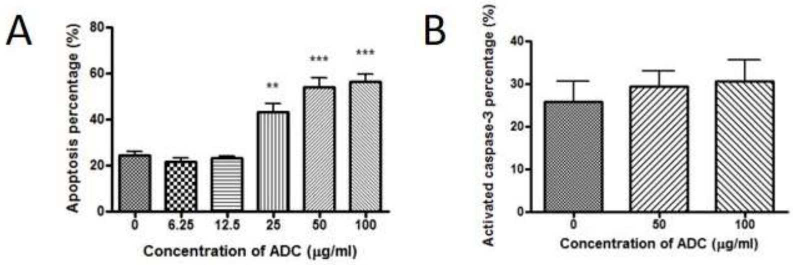

Supplementary Figure S3 ADI sensitive cancers demonstrate a significant down-regulation of ASS1 expression. Expression of ASS1 level in (A) cholangiocarcinoma (CHOL), (B) liver hepatocellular carcinoma (LIHC) and (C) colon adenocarcinoma (COAD) in the TCGA (The Cancer Genome Atlas) database.

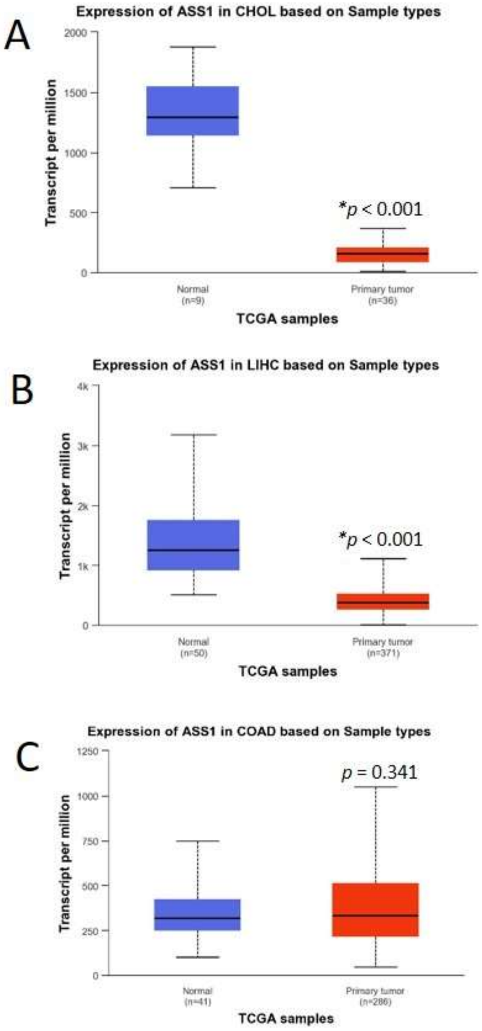

Supplementary Table S1 IC50 (ug/ml) and Maximum cytotoxicity (%) of ADC in various colorectal cancer cell lines. IC50 value is defined as the amount of ADC needed to achieve 50% inhibition of cell growth.

Cancer Type	Cell Line	IC_{50} (μg/ml)	Maximum cytotoxicity (%)
Colorectal	HCT116	12.23	84.35
	COLO 205	19.40	81.62
	LoVo	38.09	66.17
	SW1116	21.30	73.69
